# Inadequacy of nutrients and contaminants found in porridge‐type complementary foods in Rwanda

**DOI:** 10.1111/mcn.12856

**Published:** 2019-07-15

**Authors:** Silke Grosshagauer, Peiman Milani, Klaus Kraemer, Assumpta Mukabutera, Alexander Burkon, Marc Pignitter, Sebastian Bayer, Veronika Somoza

**Affiliations:** ^1^ Faculty of Chemistry, Department of Physiological Chemistry University of Vienna Vienna Austria; ^2^ Sight and Life Foundation Basel Switzerland; ^3^ College of Medicine and Health Sciences, School of Public Health University of Rwanda Kigali Rwanda; ^4^ Chemisches Institut Burkon Partnerschaft Nuremberg Germany

**Keywords:** complementary foods, food and nutrient intake, food consumption, food intake, infant and child nutrition, infant feeding

## Abstract

Child malnutrition remains persistently high in Rwanda. Complementary foods play a key role in young child nutrition. This study explores the quality and safety of complementary food products available in the Rwandan market. Ten of the most consumed porridge‐type complementary food products in Rwanda have been analysed. Mean values of macronutrient and micronutrient contents were compared against three international standards and evaluated against label claims. Mean mycotoxin, microbiological, and pesticide contamination were compared with maximum tolerable limits. Mean energy density (385 kcal/100 g) and total fat content (7.9 g/100 g) were lower than all three international benchmarks. The mean fibre content of 8.5 g/100 g was above the maximum recommended amount of Codex Alimentarius and more than double the amount claimed on labels. Mean levels of vitamin A (retinyl palmitate, 0.54 mg/100 g) and vitamin E (α‐tocopherol, 3.7 mg/100 g) fell significantly short of all three standards, whereas calcium and zinc requirements were only partially met. Average iron content was 12.1 mg/100 g. The analysis revealed a mean aflatoxin contamination of 61 μg/kg, and high mold and yeast infestation. *Escherichia coli* and pesticide residues were found, whereas no heavy metals could be quantitated. Overall, complementary food products in Rwanda show inadequate nutrient contents and high aflatoxin and microbial contamination levels. Improved regulation and monitoring of both local and imported products are needed to improve the quality and safety of complementary foods in Rwanda.

**Key messages**

- Complementary food safety and quality are an important public health concern, given their popularity in many low‐ and middle‐income countries and the vulnerability of children 6–24 months old.
- An analysis of the 10 most popular complementary foods available in the Rwandan market shows high mycotoxin, microbiological and pesticide contamination, besides deviations of actual nutrient contents from label claims and international standards.
- Standards for commercial premixed infant cereals need to be set to improve quality in food manufacturers' practices and establish independent assurance systems that enforce standards and convey the value of food safety and quality to consumers.

**Abbreviations**
CA: Codex Alimentarius
CFs: complementary foods
CFU: colony forming units
DDT: dichlordiphenyltrichlorethane
L&D: Lutter and Dewey
LICs: low‐income countries
LOD: limit of detection
SCP: Super Cereal Plus

## 1 INTRODUCTION

Complementary foods (CFs) play a key role in child nutrition and linear growth in the 6‐ to 24‐month critical window of child development (World Health Organization, 2018). For the first 6 months, infants should be exclusively breastfed to promote optimal growth, development, and health (World Health Organization, 2013). After this phase, nutritionally adequate and safe CFs should be provided in addition to continued breast feeding up to the age of 2 years to meet their evolving nutritional requirements. (World Health Organization, 2013). Fortified CFs, such as porridge mixes, with their higher nutrient density and digestibility, are a convenient source that complements breastmilk in supplying necessary nutrients at adequate levels to the growing child (Masters, Nene, & Bell, 2017). This is especially relevant for Rwanda, and in general, sub‐Saharan Africa, where malnutrition poses a serious problem (Bhutta & Salam, 2012). Although CFs are commonly considered as nutritious and safe, comprehensive quantitative data on nutrients and contaminants especially from products available in low‐income countries (LICs), where these foods play a pivotal role in older infant and young child nutrition, are rare and mostly focused on micronutrient contents (Gibbs et al., 2011; Gibson, Bailey, Gibbs, & Ferguson, 2010). A recent study (Masters et al., 2017) assessing 108 different CF products from 22 Asian, African, and European countries found significant discrepancies between label claims and actual nutrient content, as well as serious inadequacies with regard to meeting nutrient needs at 6, 9, 12, and 24 months of age, with only 22% of the products meeting requirements for iron and 21% for zinc.

A related and equally relevant issue is food contamination. Contaminants such as mycotoxins, including aflatoxins and fumonisins, and heavy metals such as lead and mercury are known to be toxic, carcinogenic, and associated with child stunting (Gleason et al., 2016; Kumar, Mahato, Kamle, Mohanta, & Kang, 2017; Magoha et al., 2016; Wu, Narrod, Tiongco, & Liu, 2011). Two recent studies conducted in Ghana (Blankson & Mill‐Robertson, 2016) and Tanzania (Kamala et al., 2017) revealed an alarmingly high exposure to above‐limit aflatoxin levels in cereal‐based CFs. In this study, a comprehensive analysis of the nutritional quality and safety of locally marketed CF brands in Rwanda has been undertaken in order to provide a basis for improving nutrient intake and reducing exposure to contaminants of older infants and young children in LICs.

## 2 METHODS

### 2.1 Study design

Ten of the highest selling brands of porridge‐type CF available in the Rwandan market, including local brands and brands from Uganda and Kenya, as well as imports from Switzerland and France were selected (Nielsen East Africa, 2017). Their main ingredients were maize, soya, sorghum, rice, and wheat. One product additionally included sesame and peanuts, whereas others listed sprat or fruits as ingredients. Vitamin, mineral, protein, carbohydrate, lipid, and contaminant concentrations were quantitatively assessed and evaluated against label claims and three international standards of age‐based requirements.

### 2.2 Data collection

Samples from two different lots of each product were purchased between November 1, 2017, and December 19, 2017, in commercial retail outlets in the Kigali area, including supermarkets, minimarkets, and “mom and pop” shops. Despite the team's best efforts, for three among the 10 products, only one batch was found, as no second batch was available on the market within a 60‐day window. Furthermore, one product was unavailable in retail and sold exclusively to institutional buyers. In total, 17 samples underwent detailed analysis.

Each sample was individually unpacked, refilled in a sealed plastic bag, and anonymized before testing.

### 2.3 Analytical methods

For quantitation of vitamin A, sample preparation was adapted from Pignitter et al (Pignitter, Dumhart et al., 2014; Pignitter et al., 2016). A volume of 1‐g porridge sample was suspended in 130‐ml ethanol (77%, *v*/v), mixed with 1‐g sodium ascorbate and 20‐ml KOH (10.7 M), and heated at 95°C under nitrogen atmosphere for 30 min using a reflux condenser. Afterwards, the mixture was cooled to 15°C and extracted thrice with 100‐ml hexane. Organic fractions were pooled, washed with water (4 × 100 ml), evaporated to dryness, and reconstituted in 1‐ml hexane prior to analysis with HPLC‐UV at 325 nm.

For the quantitation of vitamin E, a 10‐g porridge sample was extracted for 2 hr with 200‐ml ethanol at 90°C using a soxhlet apparatus. The extract was completely evaporated using nitrogen gas, reconstituted in 1‐ml isopropanol, and analysed with HPLC‐UV at 295 nm. The HPLC analysis was performed as described previously (Pignitter, Stolze et al., 2014; Zaunschirm et al., 2018).

Peroxide value was ascertained following Pignitter, Stolze, et al. (2014). A filtration step was added to ensure visibility of the equivalence point. Vitamin A, vitamin E, and peroxide analyses were conducted at the Department of Physiological Chemistry, Faculty of Chemistry, University of Vienna, Austria.

Further chemical and microbiological analyses were performed at Chemisches Institut Burkon Partnerschaft, Nuremberg, Germany. These analyses followed international and national standardized reference methods set by the German code of law for food and feed (§ 64 LFGB*), the Association of Official Analytical Chemists, the European Committee for Standardization and the Nordic Committee for Food Analysis. Chemical methods are further detailed as follows: dry matter (method no. analog L 06.00‐3*), ash (analog L 53.00‐4*), crude protein (analog L 06.00‐7*), fat (analog L 17.00‐4 mod.*), dietary fibre (L 00.00‐18*), sugars (analog L 26.11.03‐8 mod.*), nitrate and nitrite (analog L 07.00‐12*), heavy metals and minerals (L 00.00‐19* and analog L 31.00‐10 mod.*), fatty acid analysis (DGF C‐VI 10a mod. /11e), vitamin D (DIN EN 12821:2009‐08), vitamin K (DIN EN 14148:2003‐10), vitamin B_6_ (EN 14164), vitamins B_1_ and B_2_ (EN14122:2003 mod.), total folate (NMKL 111:1985), vitamin B_12_ (J.AOAC 2008, vol 91 no 4), aflatoxins B + G (analog L 23.05‐2 mod.*), aflatoxin M_1_ (analog L 01.00‐34*), ochratoxin A (analog L 15.00‐1/2 mod.*), pesticide screening (GC‐ and LC‐MS/MS, L 00.00‐115*), chlormequat and mepiquat (LC‐MS/MS, L 00.00‐76*), and Glyphosate/AMPA (LC‐MS/MS, EURL‐SRM QuPPe mod.). Mycotoxins deoxynivalenol, zearalenon, T‐2‐, and HT‐2‐toxins were analysed after extraction of 25 g homogenized sample using 100 ml of a mixture of acetonitrile/water (84:16%, *v*/v) and Ultra‐Turrax®, cleaning up the filtrate with a MultiSep‐226AflaZON® column (Romer Labs) prior to analysis with LC‐MS/MS. Fumonisins B_1_ and B_2_ were analysed after extraction of 12.5 g homogenized sample using 50 ml of an acetonitrile/methanol/water (25%:25%:50%, *v*/v) and Ultra‐Turrax® mixture, clean‐up the filtrate with a Affinimip‐FumoZON® column (Romer Labs) prior to analysis with LC‐MS/MS. Microbiological methods were total aerobic plate count (L 06.00‐18*), enterobacteriaceae (L 06.00‐24*), bact. *Escherichia* *coli* (L 01.00‐3 mod.*), yeast and mold (L 01.00‐37*), coag.‐pos. Staphylococcus (L 00.00‐55 mod.*), *Bacillus cereus* (L 00.00‐33 mod.*), sulfite‐red. clostridia (L 06.00‐32 mod.*), Listeria sp. (L 00.00‐32 mod.*), and Salmonella sp. (L 00.00‐20*). Cronobacter sp. were analysed using different selective culture solutions and culture media for differentiation.

To coherently expand the body of evidence on this topic and create a consistent literature for future meta‐analyses, we have followed the same general analytical approach of Masters et al., 2017. Thus, quantitative data obtained for nutrients and contaminants were evaluated against three international benchmarks (Table 1): Codex Alimentarius (CA; Food and Agriculture Organization/World Health Organization, 2013), Super Cereal Plus (SCP; World Food Programme, 2015), and Lutter and Dewey (L&D; Lutter & Dewey, 2003). They all set recommendations for the nutrient composition of fortified CFs, especially porridges and ready‐to‐use products, for infants aged between 6 and 23 months, assuming continued breastfeeding. Lutter and Dewey (2003) also showed separate recommendations for infants aged 6–11 months and for infants aged between 12 and 23 months. However, the analysed products were addressed to all the age groups, and therefore, we used the recommendations set for the ages between 6 and 23 months.

**Table 1:** International benchmarks for complementary food for children from 6 to 24 months (Food and Agriculture Organization/World Health Organization, 2013; Lutter & Dewey, 2003; World Food Programme, 2015)

Nutrient content (per 100 g)	CA	SCP	L&D
Energy^a^ (kcal)	400	410	440
Protein^a^ (g)	6–15	16	6–11
Fat^a^ (g)	9	9	12.7
Fiber^a^ (g)	5	2.9	
Vitamin A (retinol) (mg)	0.4	0.83–1.25	0.5
Vitamin E (α‐tocopherol) (mg)	5	8.3	10
Vitamin D (μg)	5	11.04	2–4
Vitamin K (μg)	15	30	
Vitamin B_1_ (mg)	0.5	0.2	0.36
Vitamin B_2_ (mg)	0.5	1.4	0.36
Vitamin B_6_ (mg)	0.5	1	0.44
Vitamin B_12_ (μg)	0.9	2	0.52
Folate DFE (μg)	150	110	138.3
Calcium^b^ (mg)	500	420–630	200–400
Magnesium (mg)	60		80–120
Iron^c^ (mg)	11.6	9–13.5	14
Zinc^d^ (mg)	4.1	5	8.3

Abbreviations: CA, Codex Alimentarius (Food and Agriculture Organization/World Health Organization, 2013); SCP, Super Cereal Plus (World Food Programme, 2015); L&D, Lutter and Dewey (Lutter & Dewey, 2003).

a Atwater conversion factors of 9 kcal/g of fat and 4 kcal/g of protein were used.

b Calcium: Further recommendations from Super Cereal Plus were established with 362 mg/100 g.

c Iron values of Codex Alimentarius are given for 5% dietary iron bioavailability. Requirements for Iron‐sodium EDTA: 2.5 mg/100 g and ferrous fumarate fine powder: 4 mg/100 g (Super Cereal Plus).

d Zinc values are given for medium dietary zinc bioavailability.

### 2.4 Statistical analysis

Statistical analyses were performed with Sigma Plot 11 (Systat Software Inc., Chicago, Illinois), revealing mean values and standard deviations for the selected nutrients and contaminants above the limit of detection (LOD). Data were collected in Excel before being entered into Sigma Plot.

### 2.5 Significance

Previous studies have shown either deficits in nutrient density or mycotoxin contamination in porridge‐type products to complement breastfeeding. Comprehensive studies about the quality and safety of complementary food are lacking. This study reveals deviations of actual nutrient contents of porridge‐type complementary foods available in Rwanda from label claims and international standards. Inadequacy in quality and safety of these foods available is shown due to high mycotoxin, microbiological, and pesticide contamination.

## 3 RESULTS

Our analysis revealed discrepancies between actual nutrient content and international benchmarks.

**Figure 1:**
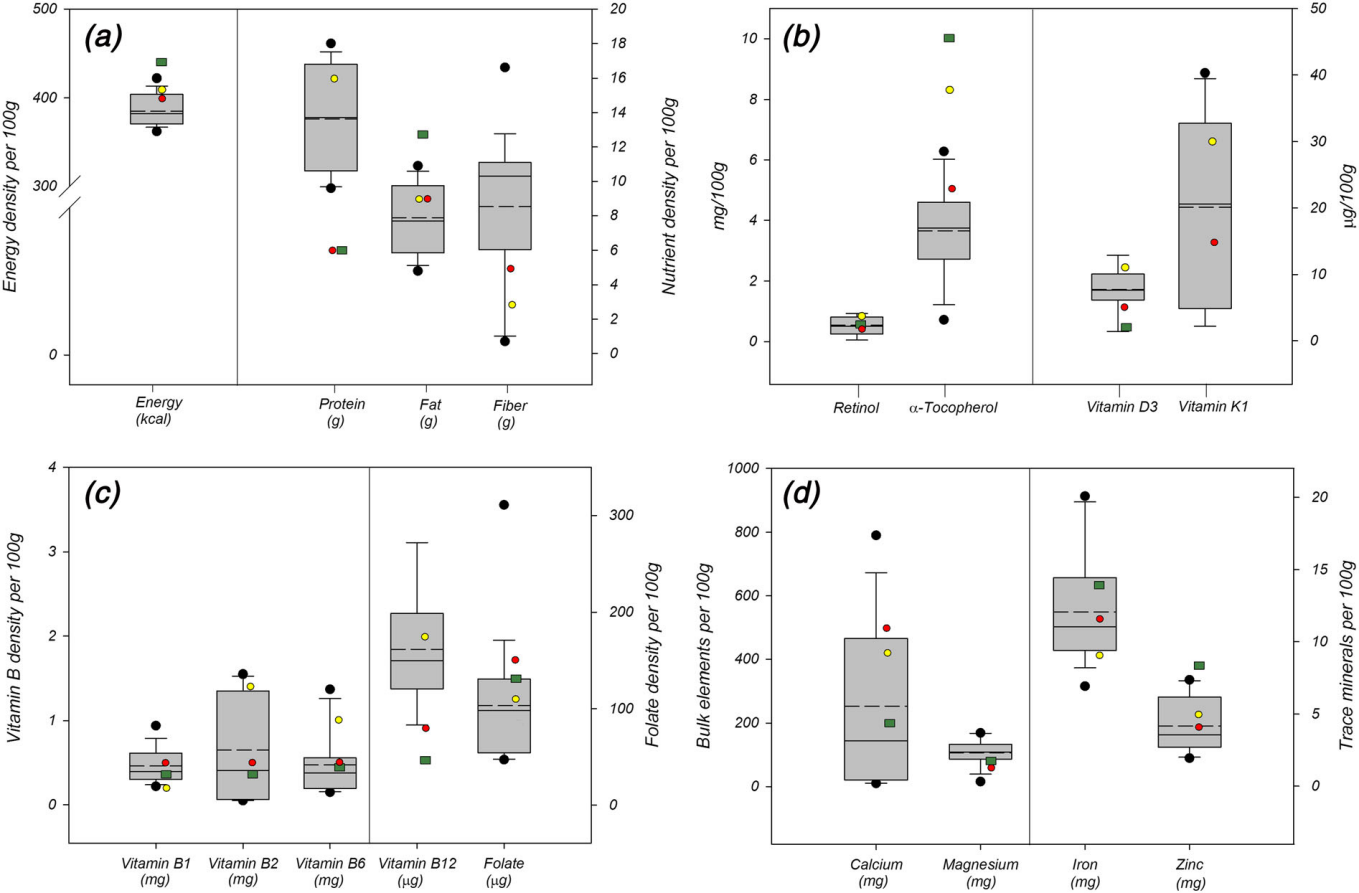
(a) Energy and macronutrient density, (b) lipid‐soluble vitamins, (c) water‐soluble vitamins, and (d) mineral density per 100 g, *n* = 17. Note: Dashed line presents mean value; red circle: Codex Alimentarius (Food and Agriculture Organization/World Health Organization, 2013), yellow circle: Super Cereal Plus (World Food Programme, 2015); green rectangle: Lutter & Dewey (Lutter & Dewey, 2003)

Average energy density of the 17 analysed samples was 385 kcal/100 g, which did not meet the requirement of 400 kcal/100 g set by CA, the least restrictive international standard for energy contents (Figure 1a). Only five out of 17 samples were above 400 kcal/100 g. On fat content, none of the products reached the L&D target of 12.7 g total fat per 100 g, and 10 samples contained less than 9‐g total fat, the minimum required by CA and SCP. For protein content, all samples contained more than 6 g of protein and, therefore, met two of the three international benchmarks (Figure 1). Although dietary fibre is important for a healthy diet, CF products with high fibre content are not recommended as they can cause children to experience satiety prematurely (Food and Agriculture Organization/World Health Organization, 2013; Food Safety Authority of Ireland, 2011). CA states that total dietary fibre should not exceed 5 g per 100 g on a dry weight basis (Food and Agriculture Organization/World Health Organization, 2013). This standard was only met by three out of 17 samples, although some labels inaccurately claimed a lower fibre content.

For lipid‐soluble vitamins, our analysis revealed an insufficient amount of retinol in the majority of the samples (Figure 1b). Vitamin A was detected in nine samples, yet only seven samples contained more than 0.4‐mg retinol suggested by CA, the least stringent benchmark for vitamin A. In the eight remaining samples, vitamin A concentration was below the LOD of 200 ng/100 g. For α‐tocopherol, only three among the 17 analysed samples contained more than 5‐mg α‐tocopherol per 100 g, the minimum set by CA (Food and Agriculture Organization/World Health Organization, 2013). Concerning vitamin E, this is the least stringent benchmark, as SCP and L&D prescribe minimum amounts of 8.3 and 10 mg, respectively. α‐Tocopherol is a homologue of vitamin E and has the highest vitamin E activity compared with β‐, γ‐, and δ‐tocopherols and tocotrienols (Kamal‐Eldin & Appelgvist, 1996). Although seven samples contained more than 5‐μg vitamin D_3_ per 100 g, the remaining 10 samples were below the requirements for CFs or below the LOD. For vitamin K, nine samples met the requirements of 15 μg/100 g set by CA. For water‐soluble vitamins, we found mean values of vitamins B_1_, B_2_, B_6_, and B_12_ above the minimum level of L&D (Figure 1c). Eight samples contained B_12_ amounts lower than the LOD of 0.25 μg/100 g. Mean total folate content of 103 μg/100 g was lower than the requirements of all three benchmarks for infants 6–23 months. Considering the CA benchmark, only one product reached the folate requirement of 150 μg/100 g.

The mean value of calcium in all 17 samples is far below the minimum requirement of 500 mg/100 g (Figure 1d). In contrast, magnesium requirements of 60 mg/100 g (Food and Agriculture Organization/World Health Organization, 2013) were reached by the majority of samples. As for iron and zinc, zinc content in 11 among the 17 analysed samples was below 4.1 mg/100 g. For iron, the mean value of the products, which reached 12.1 mg/100 g, was above the minimum 11.6 mg/100 g required by CA (Food and Agriculture Organization/World Health Organization, 2013). Average iron content was also above the least stringent benchmark (SCP; World Food Programme, 2015) of between 9 and 13.5 mg/100 g. A further recommendation for iron‐sodium EDTA and ferrous fumarate fine powder was established with 2.5 mg/100 g and 4 mg/100 g, respectively, in SCP (World Food Programme, 2015). However, only two products met the L&D iron requirements of 14 mg/100 g in the first, as well as in the second lot (Lutter & Dewey, 2003).

**Figure 2:**
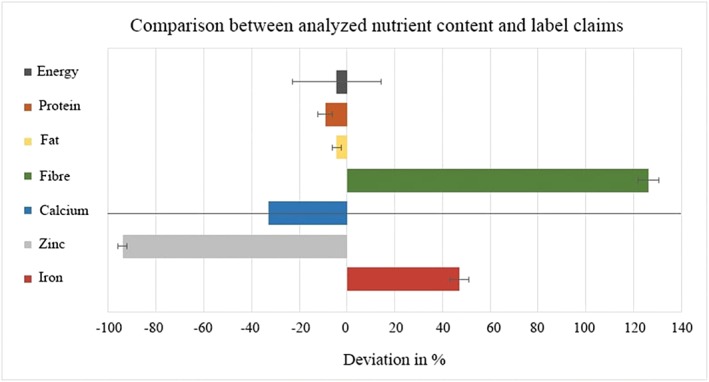
Percent deviation from label claims for quantitated levels of energy (*n* = 10), protein (*n* = 10), fat (*n* = 10), fibre (*n* = 8), calcium (*n* = 10), zinc (*n* = 9), and iron (*n* = 10). The mean values of label claims were compared against the mean values of actual contents (*n* = 17)

Furthermore, the analysis revealed discrepancies between actual nutrient content and label claims (Figure 2).

On average, products claimed energy density of 402 kcal per 100 g, though seven samples could not be evaluated as their labels provided neither energy nor nutritional content information. For fat content, a deviation of approximately 4.3% from label claim was observed. One sample claimed to contain 15.8 g fat per 100 g, whereas only 9.7 g of total fat were observed. On average, 3.8 g/100 g of fibre were claimed on labels, far below actual fibre contents of 8.5 g/100 g. One product claimed fibre content of 5 g per 100 g against more than 11 g of actual total fibre. Although labels claimed 378 mg/100 g calcium on average, actual average calcium content was 253 mg/100 g. The calcium content of one product spread substantially across batches: 499 mg/100 g in the first batch versus 790 mg/100 g of calcium in the second.

Zinc claims on six of the products averaged 70.4 mg/100 g, far above actual results. Even excluding the wildest label claim, the 9.4 mg/100 g mean claim was more than double the amount the analysis revealed.

Samples were also subjected to contaminant analyses. For heavy metals, none of the samples contained lead, cadmium, arsenic, or mercury in detectable amounts. Peroxide values were likewise below the LOD of 0.138 milliequivalent oxygen/kg. Residues of pesticides chlorpyrifos, cypermethrin, dichlordiphenyltrichlorethane (DDT), α‐endosulfan, malathion, permethrin, piperonyl butoxide, pirimiphos‐methyl, profenofos, propoxur, and chlormequat/mepiquat were detected in 12 out of 17 samples. These exceeded the maximum allowed level in Dietary Regulation §14 of the European Commission EC 396/2005 for cereal‐based foods of 0.01 mg/kg (European Commission, 2005). DDT, detected in one product, also exceeded the limit of 0.5 mg/kg for maize per European Commission EC 396/2005 (European Commission, 2005). Profenofos and chlorpyrifos limits (European Commission, 2005) were also exceeded in soya‐based products, up to 0.29 mg/kg and 0.23 mg/kg, respectively, in the worst cases.

**Figure 3:**
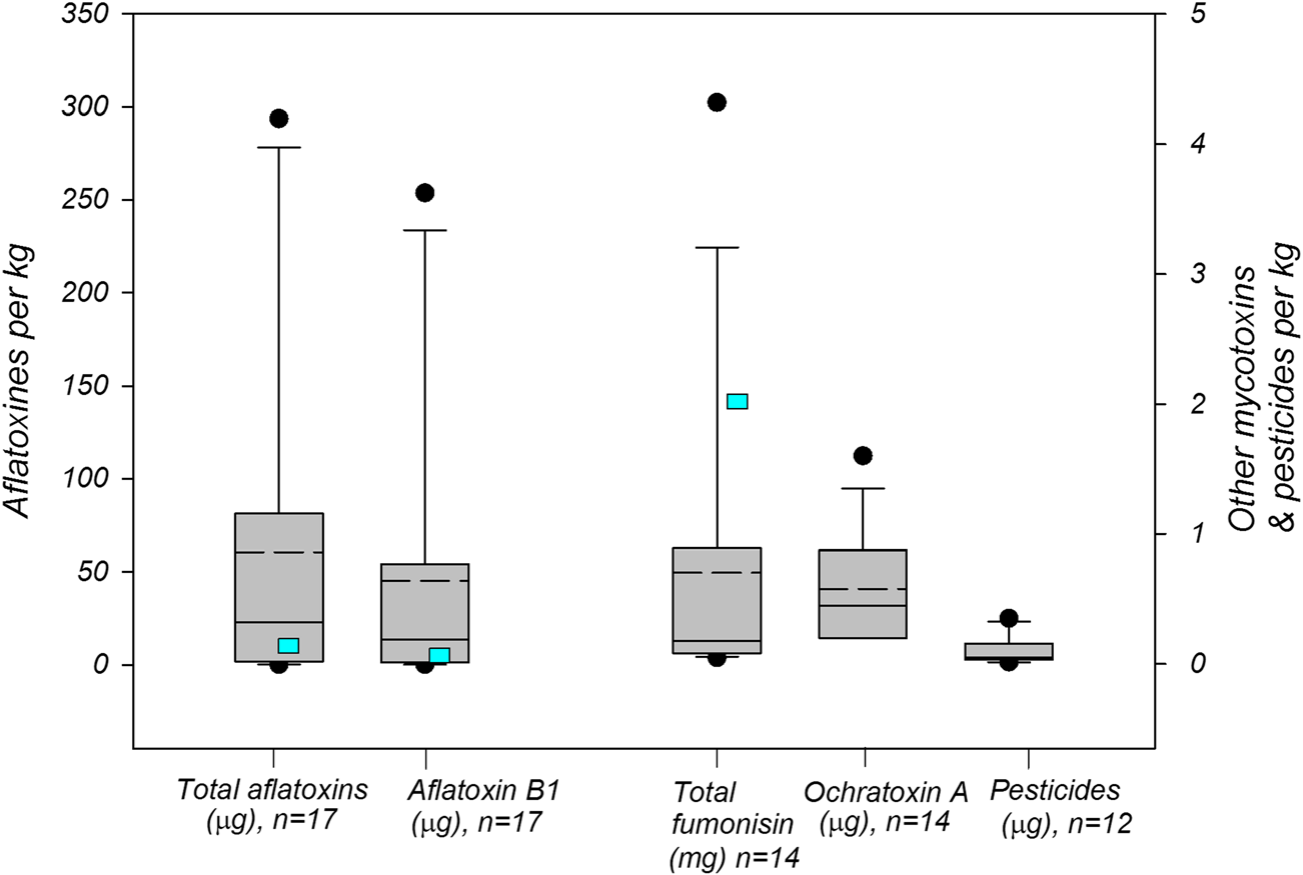
Mycotoxin and pesticide contamination per kg; dashed line presents mean value; blue rectangle: maximum tolerable limit according to Rwanda Standards Board (2016)

Alarmingly high aflatoxin contamination was found in nine of the 17 samples, with 294 μg/kg of total aflatoxin and 229 μg/kg of aflatoxin B_1_ being the highest detected values (Figure 3). This far exceeds the tolerable limits of 10 μg/kg of total aflatoxins (B_1_, B_2_, G_1_, and G_2_) and 5 μg/kg of aflatoxin B_1_ set by the Rwanda Standards Board (2016), not to mention SCP's total aflatoxin limit of 5 μg/kg. Detectable amounts of aflatoxins were also present in the remaining samples.

Fumonisins and ochratoxin A were present in 14 of the samples, and deoxynivalenol, in three samples, albeit within permissible limits of 10 μg/kg (ochratoxin A) and 0.2 mg/kg (deoxynivalenol; Rwanda Standards Board, 2016).

Severe mold and yeast contamination were detected (Table 2), reaching 49,000 cfu/g of mold and 5,100 cfu/g of yeast in the worst cases. Both should be absent according to the Rwanda Standards Board (2016). None of the 17 samples contained Salmonella, Listeria, or coagulase‐positive Staphylococci in detectable amounts. Although *E. coli* could not be quantitated in the majority of the products, one product reached 190 cfu/g in one of its samples. An average of 200 cfu/g of *Bacillus cereus* was found in five samples exceeding the LOD of 100 cfu/g.

**Table 2:** Mean values of microbiological contamination

Microbiological contamination	Number of contaminated samples	Unit	Results^a^	Limits
Bact. *Escherichia coli*	3	cfu/g	65 ± 270	Shall be absent
*Bacillus cereus*	5	cfu/g	200 ± 124	Shall be absent
Coag.‐pos. Staphylococcus	0	cfu/g	nd	Shall be absent
Yeast	1	cfu/g	5100	Absent
Mold	12	cfu/g	18,383 ± 11,320	Absent
Listeria	0	cfu/g	nd	Absent
Salmonella	0	cfu/25 g	nd	Absent

a Results are given in mean values ± confidence interval (95%).

## 4 DISCUSSION

The number of investigations of CF nutrient and contamination analysis in LICs is small. The intent of this study was to raise the quality bar for the Rwandan CF market and improve child nutrition in Rwanda. Parents feeding these cereal‐based products to their growing infants and children expect an adequate supply of nutrients that cannot be ensured with breastmilk alone. Unfortunately, the study showed discrepancies between actual nutrient content and label claims, with energy, protein, and fat content claims exceeding actuals. Conversely, dietary fibre levels were underestimated on labels. Vitamin E and iron contents were generally higher than labels indicated, whereas vitamin A, calcium, and zinc were rather overestimated on labels.

International standards such as CA, SCP, and L&D and national guidelines represent a helpful approach to optimize nutrient supply for infants if they are followed (Food and Agriculture Organization/World Health Organization, 2013; Lutter & Dewey, 2003; Rwanda Standards Board, 2016; World Food Programme, 2015). By analysing the nutrient content of 108 food products in different parts of the world, with sub‐Saharan countries among them, Masters et al. (2017) showed that nutrient requirements are not fully met by the majority of the products. Focusing on the Rwandan market in this paper, the outcomes are in accordance with previously published results and still show the same characteristics with regard to nutrient inadequacy. We used similar data visualization techniques and benchmarks, thereby enabling a direct comparison of these study outcomes with additional analysis of nine vitamins and two further minerals. Of special concern is vitamin A supply. According to the World Health Organization, approximately 6% of child deaths under the age of 5 can be attributed to vitamin A deficiency in Africa (World Health Organization, 2009a). In Rwanda, in particular, night blindness already appears in preschool aged children and is considered a moderate public health problem (World Health Organization, 2009b). Currently, 96% of children aged between 6 and 59 months are receiving two annual doses of life‐saving vitamin A (UNICEF, 2017). CFs rich in vitamin A would be a suitable approach to prevent inadequacy of this lipid‐soluble vitamin. Unfortunately, the current study reveals the need for considerable improvement of CF products in that regard.

Antinutrients such as phytates, tannins, lectins, trypsin, and chymotrypsin inhibitors can lower protein quality and digestibility and mineral absorption. In a comprehensive assessment of antinutrients in a wide range of CFs, Roos et al. (2013) found phytate at levels potentially compromising mineral absorption in processed cereal‐ and legume‐based products. Trypsin and chymotrypsin inhibitors and lectins were detected in residual quantities with unclear health effects in malnourished infants. Future work should assess both nutrients and antinutrients, but this was beyond the scope of this study.

Mycotoxin contamination, and particularly exposure to aflatoxins, was responsible for 125 deaths in Kenya in 2004 (Lewis et al., 2005). It is critical to keep food, especially products consumed by children at this very young age, safe from these contaminants, as aflatoxin B_1_ is classified as mutagenic and carcinogenic (Doi & Uetsuka, 2014; Wild, Miller, & Groopman, 2015). The present study showed that mycotoxin and microbiological contamination, as well as the presence of pesticides, represent an unacceptable risk for this vulnerable population. The problem of high aflatoxin levels lies not only in the fungal‐promoting weather conditions of Africa (Clay & Dejaegher, 1987) but also in the lack of awareness of mycotoxin contamination risks (Nishimwe, Wanjuki, Karangwa, Darnell, & Harvey, 2017).

The high susceptibility of maize products to aflatoxins in our study is consistent with existing literature and highlights the need for deliberate action to ensure food safety in infant products. Two previous studies provided information on mycotoxin levels in products available in the Kigali market (Matsiko et al., 2017; Nishimwe et al., 2017). More than 680 maize flours were analysed in the study of Nishimwe et al. (2017), showing total aflatoxin levels generally above tolerable limits set by the European Commission (4 μg/kg; European Commission, 2006), the Kenyan Government, and the United Nations World Food Programme (10 μg/kg; International Food Programme Research Institute, 2011) and the U.S. Food and Drug Administration (20 μg/kg; U.S. Food and Drug Administration, 2000) for maize products. The analysis revealed aflatoxin B_1_ levels between 8 and 24.7 μg/kg, and between 10.2 and 25.7 μg/kg in Rounds 1 and 2, respectively. Matsiko et al. (2017) analysed the aflatoxin contamination of maize and cassava flours available in the Kigali market. Unlike cassava flours, which remained under the LOD of aflatoxins, maize flours showed higher aflatoxin contamination, with 13% of the samples exceeding 5 μg/kg of aflatoxin B_1_. The authors assumed that these results might be due to higher fungal resistance in cassava plants.

The small sample size, as well as the exclusive investigation of the Rwanda market, are limitations of the current study. The focus was on a comprehensive and deep analysis, covering not only energy and nutrient density but also contamination.

In conclusion, our study revealed significant discrepancies between label claims and actual nutrient contents and between the latter and international standards. It thus highlights the need to reduce losses during production processes and enhance stability of essential micronutrients during storage under the challenging climate conditions in Rwanda and other LICs. Implementation of improved regulatory monitoring of nutrient content and contaminants for CFs in the Rwandan market and likely in other LICs will be essential to reduce child malnutrition and reach Sustainable Development Goal 2 of ending hunger, achieving food security, and improving nutrition and promoting sustainable agriculture.

## CONFLICTS OF INTEREST

Dr. Klaus Kraemer and Peiman Milani are affiliated with Sight and Life Foundation, a humanitarian nutrition think tank supported by Royal DSM. The other authors have no potential conflicts of interest to disclose.

## CONTRIBUTIONS

KK, PM, AM, MP, and VS designed the study; selected the product brands to be analysed based on the market data; acquired the product samples in Kigali; carried out vitamin A, vitamin E, and peroxide value analyses; and reviewed and revised the manuscript. SG drafted the initial manuscript. AB and SB carried out the chemical analyses and helped revising the manuscript.
